# Towards automatic text-based estimation of depression through symptom prediction

**DOI:** 10.1186/s40708-023-00185-9

**Published:** 2023-02-13

**Authors:** Kirill Milintsevich, Kairit Sirts, Gaël Dias

**Affiliations:** 1 Institute of Computer Science, University of Tartu, Tartu, Estonia; 2 Groupe de Recherche en Informatique, Image et Instrumentation (GREYC), National Graduate School of Engineering and Research Center (ENSICAEN), Université de Caen Normandie (UNICAEN), 14000 Caen, France

**Keywords:** Computational methods for mental health, Automated depression estimation, Natural language processing, Symptom network analysis, Multi-target regression

## Abstract

Major Depressive Disorder (MDD) is one of the most common and comorbid mental disorders that impacts a person’s day-to-day activity. In addition, MDD affects one’s linguistic footprint, which is reflected by subtle changes in speech production. This allows us to use natural language processing (NLP) techniques to build a neural classifier to detect depression from speech transcripts. Typically, current NLP systems discriminate only between the depressed and non-depressed states. This approach, however, disregards the complexity of the clinical picture of depression, as different people with MDD can suffer from different sets of depression symptoms. Therefore, predicting individual symptoms can provide more fine-grained information about a person’s condition. In this work, we look at the depression classification problem through the prism of the symptom network analysis approach, which shifts attention from a categorical analysis of depression towards a personalized analysis of symptom profiles. For that purpose, we trained a multi-target hierarchical regression model to predict individual depression symptoms from patient–psychiatrist interview transcripts from the DAIC-WOZ corpus. Our model achieved results on par with state-of-the-art models on both binary diagnostic classification and depression severity prediction while at the same time providing a more fine-grained overview of individual symptoms for each person. The model achieved a mean absolute error (MAE) from 0.438 to 0.830 on eight depression symptoms and showed state-of-the-art results in binary depression estimation (73.9 macro-F1) and total depression score prediction (3.78 MAE). Moreover, the model produced a symptom correlation graph that is structurally identical to the real one. The proposed symptom-based approach provides more in-depth information about the depressive condition by focusing on the individual symptoms rather than a general binary diagnosis.

## Introduction

Major Depressive Disorder (MDD) is one of the most common mental disorders, with over 300 million people being affected by it [1]. Diagnostic and Statistical Manual of Mental Disorders (DSM-5) [2] defines MDD by nine symptoms: (1) depressed mood; (2) markedly diminished interest or pleasure; (3) increase or decrease in either weight or appetite; (4) insomnia or hypersomnia; (5) psychomotor agitation or retardation; (6) fatigue or loss of energy; (7) feelings of worthlessness or inappropriate guilt; (8) diminished ability to think or concentrate; (9) recurrent thoughts of death or recurrent suicidal ideation. According to DSM-5, the diagnosis of MDD is warranted if the person has experienced at least 5 of those symptoms every day or almost every day for the last two weeks, and one of those symptoms must be either depressed mood (1) or the loss of interest (2). These diagnostic criteria indicate that behind the same diagnostic label, there can be many different symptom constellations or sub-types [3, 4].

### Background

In recent years, considerable interest has emerged in using natural language processing (NLP) and artificial intelligence (AI) techniques for inferring the mental health status of a person unobtrusively based on their speech or writing (see for instance [5, 6] for reviews). A large majority of studies have focused on predicting depression [6], which is only to be expected considering its prevalence. However, most NLP and AI-based systems have treated the task as a discrete binary classification problem [7–9], predicting the presence or absence of the diagnosis, which does not appreciate the variability of the clinical phenomena of depression.

**Fig. 1:**
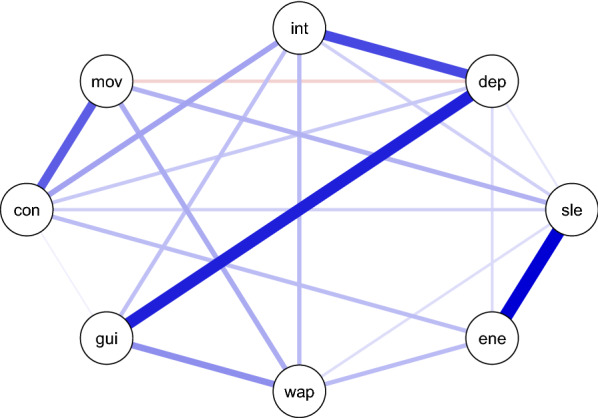
Correlation graph of symptoms computed on the training set of the DAIC-WOZ data set. Thicker edges show a stronger correlation. Blue edges show a positive correlation, and red edges show a negative correlation. The nodes represent the following symptoms: *int*: markedly diminished interest or pleasure; *dep*: depressed mood; *sle*: insomnia or hypersomnia; *ene*: fatigue or loss of energy; *wap*: increase or decrease in either weight or appetite; *gui*: feelings of worthlessness or inappropriate guilt; *con*: diminished ability to think or concentrate; *mov*: psychomotor agitation or retardation

Although psychiatric diagnostic systems like DSM-5 still mostly operate with categorical diagnoses, there is a shift towards richer representations of psychiatric syndromes that can take into account the dimensional and heterogeneous nature of the clinical pictures of the same psychiatric diagnosis. One particular approach that is gaining attention concerns symptom network analysis (SNA) [10, 11]. According to the SNA, the symptoms of mental health disorders are not indicators of an underlying disease (an assumption of a traditional medical model), but it rather views the disorder itself as a causal system of interacting symptoms. The advantage of the SNA is that it also provides a natural way of analyzing and modeling the comorbidity between different disorders (see, for instance, [12] and [13] for examples), which is a norm rather than an exception for mental disorders. Depression, in particular, has been studied quite a lot from the perspective of SNA [14–16]. One way of depicting the SNA graphically is to use correlation graphs, such as the one shown in Figure 1. Although the symptom graph constructed based on correlations does not show the causal links between symptoms^1^, it does show the strength of the co-occurrence relations between each pair of symptoms. The SNA view of the diagnosis prescribes a more thorough analysis of specific depression symptoms in clinical studies [17]. Thus, it seems only natural to extend the research based on NLP and AI to reflect these advances in psychiatry and start focusing on predicting the presence or degree of particular depression symptoms instead of the categorical diagnosis.

Developing predictive systems for mental health comes with the challenge of obtaining clinical data for training models. Getting patient speech or textual data is challenging due to ethical and legal reasons. Therefore, many studies have resorted to analyzing social media data [6] or other auxiliary data resources. In order to train predictive models, the clinical data needs to be supplied with diagnostic labels. One way of acquiring labels is asking people to fill in self-report questionnaires assessing the presence and/or severity of depression symptoms [18]. There are several questionnaires that assess the presence or absence of MDD based on depression symptom severity, such as the Beck Depression Inventory (BDI) [19], Hamilton Rating Scale for Depression (HRSD) [20], and Patient Health Questionnaire (PHQ) [21]; the last one shadowing the symptoms defined by DSM-5.

### Problem

Previous approaches that have used the data with self-report questionnaire scores typically obtain the labels by first summing the scores of all the questions and then dichotomizing the sum at a predefined cutoff point, which results in a binary diagnostic status. This approach, however, has several problems. First of all, using the sum of scores of these questionnaires might not be a good basis for establishing the diagnostic status of a person [17], as identical labels can hide a set of very different symptom severity values. Second, the difference in depression level between two persons with the same label can end up being larger than the difference between two persons with differing labels. For instance, in the boundary cases, one person with the non-depressed label might have obtained a sum-score of only one point lower than another person who was labeled as depressed. At the same time, two people, both having the depression label, might have a very large score difference—one having a sum-score near the cutoff and the other one at the high end of the scale. These within- and between-group characteristics can make it hard for systems to learn true patterns about depression.

### Methods

To create a model which is able to produce more fine-grained predictions we treat automatic depression prediction as a multi-target regression problem, predicting the severity score of each symptom from a common interview representation. We show that predicting each symptom individually not only gives more insight into a person’s mental state but also allows to infer the binary, 5-class, and regression scores with gains in performance in most of the experimental configurations.

In this paper, we use DAIC-WOZ [22], a data set widely used for automatic depression prediction. It consists of interviews between a person and a human-controlled virtual assistant, Ellie. Each interview has facial features from the video, audio recording, and text transcription. Each interview is also accompanied by the answers to the PHQ-8 screening questionnaire—an eight-symptom version of the PHQ, which does not include the suicidality/self-harm question from the depression diagnostic criteria. The data set is relatively small, featuring only less than 200 interviews. However, it is closer to the domain of clinical interviews than the social media data often used for developing predictive systems for mental health. Even though the DAIC-WOZ data set provides severity scores for each individual question, previous works using this data for developing automated systems have predicted either a binary label, i.e., depressed or non-depressed, [7–9, 23], or a regression score based on the total sum of individual PHQ-8 question scores [23–27]. Few other studies have discretized the range of PHQ-8 scores into five categories and have thus predicted a label within a set of five possible classes, i.e., no symptoms, mild, moderate, moderately severe, severe depression [25, 28].

### Contributions

Our goal in this study is twofold. First, we want to highlight the importance of the advances in the clinical field when developing NLP and AI-based mental health prediction models. In particular, we want to emphasize the turning away from the medical latent disease model with its categorical diagnostic predictions and more toward dimensional and symptom-level analyses. Second, we aim to demonstrate that by adopting the symptom-level prediction, the models do not lose accuracy also on the categorical diagnosis level and can add a more fine-grained representation of the clinical picture for each person, thus better capturing the heterogeneity of the clinical phenomena.

## Related work

Most studies on MDD that make use of NLP and AI methods over clinical data have been developed over the DAIC-WOZ [22] data set, although some marginal works have been carried out on the General Psychotherapy Corpus (GPC) from Alexander Street Press [8, 29]. In particular, the GPC comprises a large collection of transcripts of patient–provider conversations, but as it is not easily available^2^, most researchers have been focusing on the DAIC-WOZ for reproducibility purposes. DAIC-WOZ is a multimodal data set containing interviews accompanied with facial features from the videos, audio recordings, and text transcriptions. Therefore, various previous works have tackled the multimodal aspect of this data set.

In our work, we only make use of the textual transcriptions; thus, we limit our review to those works that have also focused on the textual modality of this data set. One line of work has concentrated on exploring various neural network architectures to best model the interviews, including hierarchical attention-based networks [7] and deep neural graph structures [27]. Other studies have experimented with multi-task modeling, aiming to improve the performance by simultaneously predicting both binary diagnostic and the overall depression severity regression scores [24]. Finally, some studies have explored the utility of enriching the models with additional, in particular affective, information from external sources. In this regard, Xezonaki et al. [8] experimented with explicitly modeling the affective features of words extracted from various affective lexicons. Qureshi et al. [25] employed an additional emotion data set and experimented with a multi-task classification model to concurrently predict both the depression severity level of the DAIC-WOZ data and the emotional intensity of the emotion data set.

All these previous studies concerning predicting depression based on clinical data of patient–therapist interviews have developed categorical models to predict the binary, multi-class, or continuous diagnostic status. The only previous work we are aware of that has used the DAIC-WOZ data set for symptom prediction is by Delahunty et al. [30]. However, as their focus was on modeling the comorbidity between depression and anxiety, they only predicted the two main depression symptoms (lowered mood and loss of interest) instead of the full symptom profile. Next, we will review some studies based on social media data that have adopted symptom prediction either instead of or for aiding the diagnostic classification.

Studies based on social media data have used Twitter [31, 32], Reddit [33] or other depression-related internet forums [34–36] as their data source. Even though these works collect their data from public sources, the data sets themselves are not publicly available. Some authors [31–33], however, stated that their data sets can be accessed by other researchers who agree to follow the ethical guidelines put forward by the corresponding authors. A challenge with working with social media data is obtaining the labels necessary for training classification models. One option is to manually label the symptoms in the data. This approach was adopted by Yadav et al. [31], who annotated the symptoms in tweets using a mental health lexicon constructed by mental health professionals. The main focus of this work was to use an auxiliary classification task to detect figurative speech that might be used to express symptoms and can be hard to detect via lexicon lookup. Yao et al. [34] analyzed a Chinese depression forum for depression symptom prediction. Their work aimed to develop a comprehensive annotation scheme for a list of symptoms that goes beyond the diagnostic symptoms of DSM-5. Davcheva et al. [36] developed a symptom-based classification system using internet forum data. The data were manually annotated with the symptom lexicon constructed based on DSM-5 symptom descriptions and topic modeling. The overall goal of the model was to provide a categorical diagnosis based on the predicted symptoms. Several diagnoses were addressed in this work, also targeting schizophrenia and attention deficit hyperactivity disorder in addition to depression.

An alternative to manual labeling is to use lexicons or rules to automatically extract the symptom mentions. This approach was adopted by Karmen et al. [35], who used lexicons to detect the mention of symptoms in the posts of an internet forum. The goal of their work was to simulate assessing the depression severity score with a self-report assessment measure by aggregating the symptom scores with the frequency of symptom mentions. Similarly, Yazdavar et al. [32] used a lexicon-based approach on tweets to compile user-specific depression lexicons and adopted a semi-supervised topic modeling approach to model the symptom progression over time. Recently, Nguyen et al. [33] adopted Reddit data to train models to predict depression diagnosis grounded in PHQ-9 symptoms. In their work, the symptoms were automatically annotated using manually constructed symptom patterns. The symptom mentions found that this way thus serves as weak labels that were used to constrain the model to predict the binary diagnosis.

## Method

While previous works that tackle patient–therapist interviews have been developing automated systems that either predict a categorical label or a regression score, the SNA approach aims at scoring each symptom individually. As a consequence, shifting to the paradigm of multi-target regression architectures is necessary. In this section, we overview the DAIC-WOZ data set and present the experimented learning architectures.

### Data

The DAIC-WOZ data set contains 189 clinical interviews in a dialog format. Each interview has two actors: the virtual assistant Ellie and a participant. The utterances of Ellie come from a predefined set of prompts, although the exact subset of prompts and their ordering can vary for each interview. The data set is distributed in pre-determined splits, such that 107 interviews are used for training, 35 for validation, and 47 for testing (see Table 1). Each interview in the data set is accompanied with a PHQ-8 assessment, which consists of eight questions inquiring about diagnostic depression symptoms. Each question is scored from 0 to 3, and the total PHQ score, which is the sum of the scores of all eight questions, ranges from 0 to 24. According to the standard cutoff score of 10, the interviews can be divided into diagnostic classes, where the subjects whose PHQ-8 total score is less than 10 are considered non-depressed, and those whose score is at least 10 are categorized as depressed. Based on the total score, the interviews can be further divided into five classes according to the depressive symptom severity [21]. From the overall layout of the DAIC-WOZ data set shown in Table 1, it is evident that the classes are imbalanced, and the imbalance is even stronger in the high PHQ score range.

**Table 1:** Number of interviews for each depressive symptom severity category in the DAIC-WOZ data set, distributed by train, validation and test sets

Depression severity	Data split		
	Train	Validation	Test
No symptoms [0..4]	47	17	22
Mild [5..9]	29	6	11
Non-depressed Total	76	23	33
Moderate [10..14]	20	5	5
Moderately severe [15..19]	7	6	7
Severe [20..24]	4	1	2
Depressed Total	31	12	14
Total	107	35	47

### Model

To efficiently encode the interviews, we employed a hierarchical architecture [37]. Since we aim at predicting scores for individual symptoms, we adopted a prediction head that produces eight regression outputs, effectively making it a multi-target regression model.

**Fig. 2:**
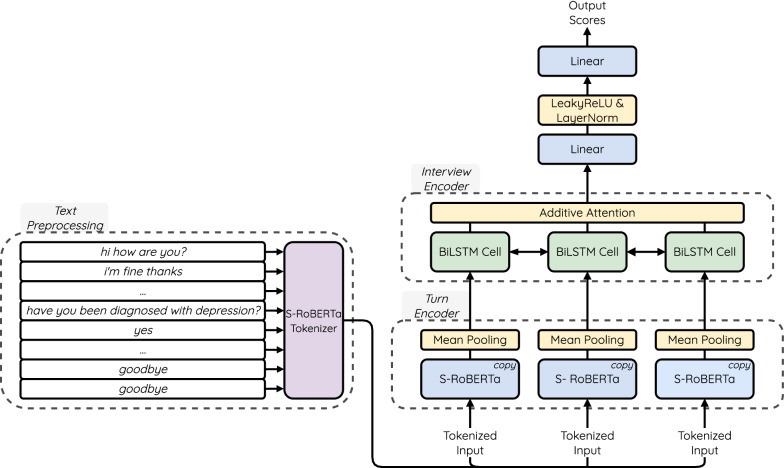
Overview of the model. On the turn-level, the same instance of S-RoBERTa is used to encode each turn. Mean Pooling is the operation that averages all the token representations output by S-RoBERTa

The model has two encoders: ${{\textbf {Enc}}}^{{\textbf {turn}}}$ and ${{\textbf {Enc}}}^{{\textbf {int}}}$. Figure 2 shows an overview of the model. First, the dialog turn encoder ${{\textbf {Enc}}}^{{\textbf {turn}}}$ encodes each interview $D = \{t_1, \dots , t_{n-1}, t_n\}$, where $t_i = \{w_1^i, \dots , w_{m-1}^i, w_m^i\}$ is a dialog turn and $w_j^i$ is a jth token in turn $t_i$, on the word level, producing an embedding ${\varvec{h}}_i^{{\textbf {turn}}}$ for each turn (1). Then, the dialog turn embeddings are processed on a higher level of the hierarchy with the interview-level encoder ${{\textbf {Enc}}}^{{\textbf {int}}}$ to produce the interview representation ${\varvec{h}}^{{\textbf {int}}}$ (2). Finally, the interview embedding is passed to a feed-forward network that maps the interview representation to a label vector $\varvec{{\hat{l}}} = [{\hat{l}}_1, {\hat{l}}_2, \dots , {\hat{l}}_7, {\hat{l}}_8]$  (3, 4, 5), where each predicted label ${\hat{l}}_k \in [0, 3]$ represents a symptom score for a corresponding question in PHQ-8. The feed-forward classifier consists of two linear layers ($W_1, W_2$) with biases (${\varvec{b}}_1, {\varvec{b}}_2$), with a LeakyReLU activation function and a LayerNorm layer [38] in-between.

1 $$\begin{aligned} {\varvec{h}}_i^{{\textbf {turn}}}= & {} {\textbf {Enc}}^{{\textbf {turn}}}(t_i) \text { for } i = 1, \dots , |D| \end{aligned}$$

2 $$\begin{aligned} {\varvec{h}}^{{\textbf {int}}}= & {} {\textbf {Enc}}^{{\textbf {int}}}(\{{\varvec{h}}_1^{\text {turn}}, \dots , {\varvec{h}}_{|D|}^{\text {turn}}\}) \end{aligned}$$

3 $$\begin{aligned} {\varvec{z}}'= & {} \text {LeakyReLU}({\varvec{h}}^{\text {int}}W^T_1 + {\varvec{b}}_1) \end{aligned}$$

4 $$\begin{aligned} {\varvec{z}}= & {} \text {LayerNorm}({\varvec{z}}') \end{aligned}$$

5 $$\begin{aligned} \varvec{{\hat{l}}}= & {} {\varvec{z}}W^T_2 + {\varvec{b}}_2 \end{aligned}$$

The word-level turn encoder ${{\textbf {Enc}}}^{{\textbf {turn}}}$ uses a distilled RoBERTa-based model from the SentenceTransformers (S-RoBERTa)^3^. SentenceTransformers is a collection of pre-trained Transformer-based language models that have been tuned to produce better sentence embeddings  [39]. RoBERTa is a Transformer-based language model which has been pre-trained on a large collection of common-domain corpora for the masked language modeling (MLM) task [40]. During MLM pre-training, some of the input tokens are masked, and the model’s objective is to predict the token that has been masked [41]. Finally, in SentenceTransformers, the model is further fine-tuned on the sentence similarity task, where the sentence embedding is produced by averaging all its respective token embeddings [39]. Furthermore, the S-RoBERTa model used in our experiments has been distilled. Knowledge distillation is a process of training a smaller student model which learns to copy the larger pre-trained teacher model [42]. Distilled models keep most of the capabilities of their full-sized counterparts while being almost twice as small and fast. Decreasing the computational complexity of our model is crucial due to the fact that all turns of the interviews have to be processed in parallel, i.e., several copies of ${{\textbf {Enc}}}^{{\textbf {turn}}}$ are created, and their respective computational graphs stored during training. The turn-level interview encoder ${{\textbf {Enc}}}^{{\textbf {int}}}$ deploys a single layer BiLSTM with a hidden dimension of 300 and an additive attention layer on top of it.

As a training objective for the symptom prediction task, the Smooth $L_1$ loss [43] was used, which is defined as in (6) for multi-target regression:

6 $$\begin{aligned} \text {Smooth}_{L_1}(\varvec{{\hat{l}}}, {\varvec{l}}) = \frac{1}{K}\sum ^K_{k=1} \text {Smooth}_{L_1}({\hat{l}}_k, l_k) \end{aligned}$$

where ${\hat{l}}_k$ and $l_k$ are the predicted and true scores for the *k*th symptom respectively, $K=8$ is the number of symptoms, and with

7 $$\begin{aligned} \small {\text {Smooth}}_{L_1}({\hat{l}}_k, l_k)={\left\{ \begin{array}{ll} 0.5({\hat{l}}_k - l_k)^2, &{} \quad {\text {if} } |{\hat{l}}_k - l_k| < 1 \\ |{\hat{l}}_k - l_k| - 0.5, &{} \quad {\text {otherwise}} \end{array}\right. } \end{aligned}$$

Since distinct random seeds can lead to substantially different results [44], each model was trained five times using different random seeds, and the average of the five runs is reported. Each model was trained for 200 epochs using AdamW optimizer with the learning rate of $3e^{-5}$ and a linear warm-up scheduler. A model checkpoint was saved after each epoch, and the checkpoint with the highest micro-averaged F1-score on the development set was chosen as the final model.

### Baseline models

To provide some validity to the symptom prediction approach, we compare the results of our model to three baseline tasks adopted in previous works: 1) binary diagnostic classification, where a patient is said to be depressed if their PHQ-8 score is at least 10, and non-depressed otherwise, 2) multi-class classification into five classes with differing severity as depicted in Table 1, i.e., no symptoms, mild, moderate, moderately severe and severe depression, and 3) depression severity prediction modeled as PHQ-8 total score regression ranging from 0 to 24.

The outputs of our multi-target regression model predicting symptom scores can be recast to a suitable format for these three tasks. For the depression severity prediction task, the symptom scores are summed up to give the estimate of the final PHQ-8 value. For the binary and multi-class classification tasks, the summed total score can be converted either into a binary label at a cutoff of 10 for the binary diagnostic classification or converted into five classes for the multi-class classification, such that [0..5) stands for no symptoms, [5..10) mild, [10..15) moderate, [15..20) moderately severe and [20..24] severe depression estimate.

For comparison, we train three baseline models that predict the three tasks directly, i.e., the model predicts one of two classes for the binary diagnostic prediction, one class out of five for the multi-class severity prediction, and a continuous score for the total depression severity regression. All baseline models use the same hierarchical architecture shown in Fig. 2; only the output layer of the feed-forward classifier network is different. Whereas the output layer for the symptom prediction model has multiple regression heads, the depression severity prediction model has a single regression head, and the models for the binary and the multi-class classifiers have a classification head that predicts one of the two or five classes, respectively.

### Evaluation

For evaluating the regression tasks (symptom scores regression and PHQ-8 total score regression), we use the mean absolute error (MAE) as defined in equation  (8), where $y_i$ is the correct PHQ-8 score, and $\hat{y_i}$ is the predicted PHQ-8 value, which in case of the symptom prediction model is obtained by summing up all the predicted symptom scores. *N* is the number of interviews in the evaluation set.

8 $$\begin{aligned} \text {MAE} = \frac{\sum ^N_{i=1}|{\hat{y}}_i - y_i|}{N} \end{aligned}$$

In order to better take into account the imbalance in scores and especially the scarcity of interviews with higher PHQ-8 total score values, we also use a macro-averaged version of the MAE ($ma\text {MAE}$), where the MAE is first computed separately for each class/score range, and then the resulting MAE-s are averaged. The computation is defined in Eq. (9), where *C* is the set of classes, $\text {MAE}^{c}$ denotes the MAE for the class *c*.

9 $$\begin{aligned} ma\text {MAE} = \frac{\sum _{c\in C} \text {MAE}^{c}}{|C|} \end{aligned}$$

For evaluating the classification tasks, we use the micro-averaged F1-score ($miF_1$) and the macro-averaged F1-score ($maF_1$) defined in Eqs. (10) and (11) respectively. For computing the precision and recall for the $miF_1$, the true positive, false positive, and false negative counts are accumulated over all classes. For $maF_1$, the class-specific F1-score $F_1^{c}$ is first computed for each class *c* separately from the class-specific precision and recall measures, and then the F1-scores for all classes are averaged.

10 $$\begin{aligned} miF_1= 2 \cdot \frac{\text {precision} \cdot \text {recall}}{\text {precision} + \text {recall}} \end{aligned}$$

11 $$\begin{aligned} maF_1= \frac{\sum _{c \in C} F_1^{c}}{|C|} \end{aligned}$$

## Results

In this section, we present the results of the multi-target architecture compared to baselines for the binary, multi-class, and regression tasks. We then show the performance of our method for each symptom individually and illustrate the symptom-based decisions for the binary and multi-class cases with radar plots. Finally, we present the results of the symptom network analysis based on non-dynamic data.

### Comparison to baselines

The top section of Table 2 shows the comparison of our Symptom Prediction model to the three baselines outlined in “3.3” section—the Binary Diagnostic model, the 5-class Severity prediction model, and the PHQ-8 Severity prediction model. Overall, the Symptom Prediction model performed better or in the same range compared to the baseline models in all evaluation tasks. In particular, the Symptom Prediction model performed considerably better than other models when evaluated on the Binary Diagnosis and the PHQ-8 Score Severity evaluation tasks. On the 5-Class Severity evaluation task, the 5-Class Severity classification model that was explicitly trained to predict these five severity classes performed better on the micro-F1 evaluation score, while on the macro-F1 evaluation score, which weighs all classes equally, both models performed similarly. We also noticed that the PHQ-8 Score Severity model, which was trained to predict the total PHQ-8 score, performed considerably worse than other models on both classification tasks.

The bottom part of Table 2 shows the results of the previous works on DAIC-WOZ data for comparison. All these works have used only text modality as input, as is also the case in our work. Overall, the Symptom Prediction model shows results that are in a similar range compared to previously published results. The only notable exception is the 5-Class Severity Evaluation task, where Qureshi et al. [25] obtained considerably higher results.

Table 3 shows the results on the development set that was used for selecting the final model. Slight overfitting on the development set can be observed for the Binary Diagnosis model. The standard deviations of the reported scores for the development set were higher than for the test set. Finally, the Symptom-based Diagnosis model was more robust than the rest of the baseline models.

**Table 2:** Experimental results on the test set of the DAIC-WOZ data set

Model	Binary Diagnosis Eval		PHQ-8 Score Severity Eval		5-Class Severity Eval	
	$miF_1 \pm \sigma$	$maF_1 \pm \sigma$	$\text {MAE} \pm \sigma$	$ma\text {MAE} \pm \sigma$	*miF*1-5c $\pm \sigma$	*maF*1-5c $\pm \sigma$
Binary Diagnosis	0.719 ± 0.016	0.701 ± 0.010	–	–	–	–
5-Class Severity	0.711 ± 0.026	0.683 ± 0.024	–	–	**0.468** ± 0.023	**0.270** ± 0.025
PHQ-8 Score Severity	0.681 ± 0.019	0.584 ± 0.024	5.03 ± 0.09	5.69 ± 0.12	0.289 ± 0.029	0.135 ± 0.014
Symptom Prediction	**0.766** ± 0.023	**0.739** ± 0.025	**3.78** ± 0.13	**4.19** ± 0.13	0.426 ± 0.014	**0.270** ± 0.019
HCAN [7]	–	0.630	–	–	–	–
HAN+L [8]	–	0.700	–	–	–	–
ASP MT. DLC+DLR+EIR [25]	–	–	3.69	–	0.600	–
HCAG-T [23]	–	0.770$\ddag$	3.73$\ddag$	–	–	–
SGNN [27]	–	–	3.76	–	–	–

Top Section: results of our model and the baselines. All models were run five times with different seed values, and the average values with standard deviation are presented; *miF*1-5c (resp. *maF*1-5c) stands for the 5-class micro-averaged F1-score (resp. macro-averaged F1-score). Bottom Section: previously published results on the same DAIC-WOZ test set using only text modality; all results are given for the best model and not based on the average performance of several runs.

Bold values indicates the best results for each model

$\ddag$ indicates that the results are given for the validation set only

**Table 3:** Experimental results on the development set of the DAIC-WOZ data set

Model	Binary Diagnosis Eval		PHQ-8 Score Severity Eval		5-Class Severity Eval	
	$miF_1$ $\pm \sigma$	$maF_1$ $\pm \sigma$	$\text {MAE}$ $\pm \sigma$	$ma\text {MAE}$ $\pm \sigma$	*miF*1-5c $\pm \sigma$	*maF*1-5c $\pm \sigma$
Binary Diagnosis	**0.806** ± 0.031	**0.798** ± 0.031	-	-	-	-
5-Class Diagnosis	0.739 ± 0.049	0.713 ± 0.058	-	-	**0.503** ± 0.049	0.237 ± 0.017
PHQ-8 Score Diagnosis	0.600 ± 0.030	0.507 ± 0.026	5.51 ± 0.06	6.01 ± 0.08	0.255 ± 0.024	0.159 ± 0.018
Symptom-based Diagnosis	0.752 ± 0.035	0.719 ± 0.047	**3.61** ± 0.12	**4.11** ± 0.18	0.442 ± 0.106	**0.286** ± 0.063

All models were run five times with different seed values, and the average values with standard deviation are presented; *miF*1-5c (resp. *maF*1-5c) stands for the 5-class micro-averaged F1-score (resp. macro-averaged F1-score). Bold values indicates the best results for each model

### Symptom prediction analysis

Next, we will look at the performance of the Symptom Prediction model for each symptom separately. The performance of each symptom was evaluated with both MAE and micro- and macro-averaged F1-scores. To compute the F1-scores, the predicted symptom scores were converted into binary labels with a cutoff of 1.5 points, such that scores lower than 1.5 were considered as symptom absent (negative class), and the scores starting from 1.5 were considered as symptom present (positive class).

While MAE is generally an effective and easily interpretable metric for evaluating regression tasks, it can give artificially low error scores when the data set is imbalanced, and the model tends to predict scores close to the mean value. Relative root mean square error (RRMSE) [45] can give a better view of the performance in those cases, as it penalizes more the model that tends to predict scores close to the mean value of the training set. RRMSE is defined in Eq. (12)

12 $$\begin{aligned} RRMSE = \sqrt{\frac{\sum ^N_{i=1}({\hat{y}}_i - y_i)^2}{\sum ^N_{i=1}(y_i - {\bar{y}})^2}} \end{aligned}$$

where ${\bar{y}}$ is the mean score of the training set, $\hat{y_i}$ is the model’s prediction, and $y_i$ is the correct score. RRMSE is a normalized measure where desirable values lie in the range of $[0\dots 1)$. RRMSE value 1 means that the evaluated model is equivalent to a naive model that always predicts the mean score of the training set, and the RRMSE value greater than 1 shows that the evaluated model is even worse than predicting the average score.

**Table 4:** Test scores for each symptom

Symptom	MAE $\pm \sigma$	RRMSE $\pm \sigma$	*miF*1 $\pm \sigma$	*maF*1 $\pm \sigma$
No interest	0.529 ± 0.047	0.877 ± 0.067	0.800 ± 0.024	0.669 ± 0.043
Depressed mood	0.550 ± 0.027	0.733 ± 0.022	0.821 ± 0.019	0.729 ± 0.024
Insomnia or hypersomnia	0.753 ± 0.073	0.805 ± 0.060	0.774 ± 0.055	0.757 ± 0.047
Feeling tired	0.638 ± 0.031	0.816 ± 0.030	0.745 ± 0.030	0.709 ± 0.035
Eating too little or too much	0.811 ± 0.049	0.972 ± 0.064	0.762 ± 0.035	0.685 ± 0.026
Feeling of being a failure	0.620 ± 0.018	0.796 ± 0.012	0.817 ± 0.024	0.779 ± 0.021
Problems with concentrating	0.830 ± 0.040	0.878 ± 0.012	0.681 ± 0.034	0.557 ± 0.029
Moving too slowly or too fast	0.438 ± 0.022	0.976 ± 0.035	0.936 ± 0.000	0.484 ± 0.000

All models were run five times with different seed values, and the average values with standard deviation are presented. For computing the F1 scores, the predicted scores were binarized, such that the scores $< 1.5$ were treated as negative class instances, and the scores $\ge 1.5$ were treated as positive class instances

Table 4 shows the symptom prediction performances. First of all, one can observe that the scores for the two main depression symptoms—depressed mood and lack of interest—are among the most accurately predicted ones across all evaluation measures; this indicates that those symptoms can be inferred sufficiently well from the interview texts. Similarly, symptoms related to sleep and feeling of being a failure show good performance relative to the other symptoms according to all measures. According to MAE and $miF_1$, the most accurately predicted symptom is movement related, but this is misleading. In our sample, the moving symptom has a relatively low score for most participants, biasing the model towards always predicting low scores. Indeed, the RRMSE score reveals that most of the predictions were close to the average value for this symptom in the data set. Furthermore, a high $miF_1$ score and a low $maF_1$ score show that the model mostly predicts scores in a very similar range—in our case, it is the symptom score in the lower end of values that will be binarized into the negative, i.e., symptom absent, class.

**Fig. 3:**
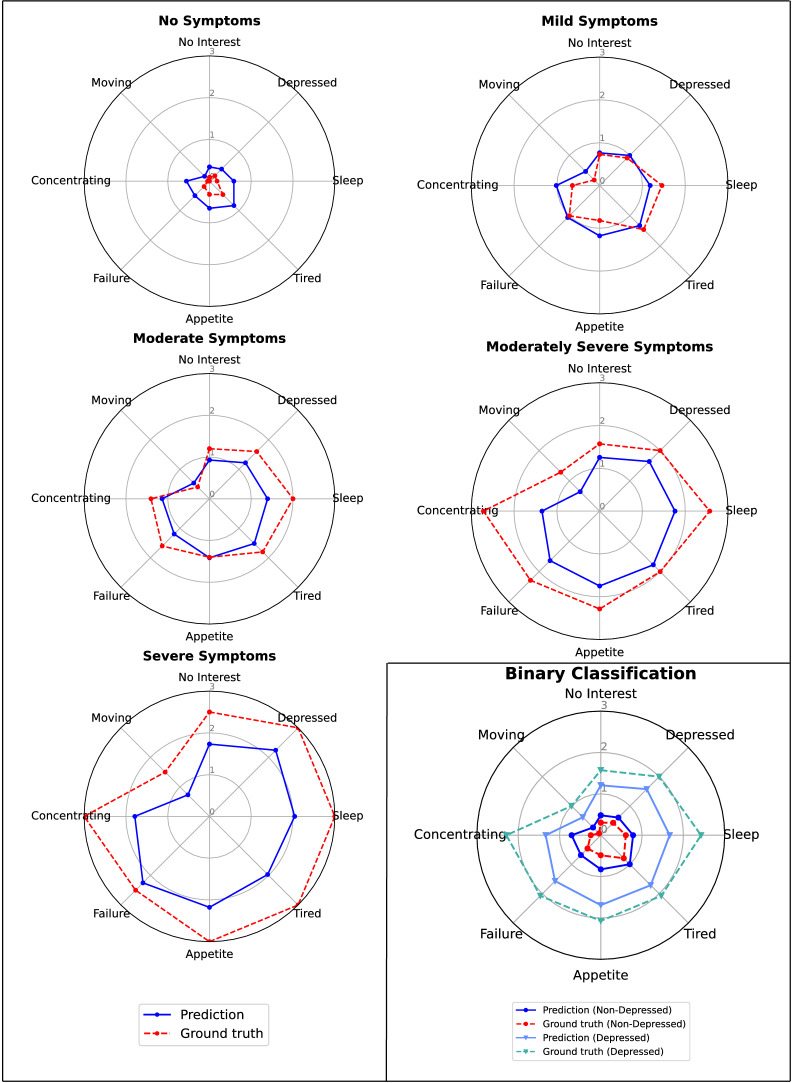
Averaged predictions and ground truth symptom scores for the test set for five fine-grained classes and binary classification. Predictions are averaged across five models trained with the same parameters and different seed values

Figure 3 shows a graphical view of the symptom predictions against the ground truth symptom scores averaged for the five-class depression severity scale (the main view) and non-depressed and depressed participants (bottom-right corner). The overall shape of the predictions generally follows the one of the ground truth scores for all groups. However, the model tends to predict scores closer to moderate ranges, thus overestimating the scores for non-depressed participants and underestimating for moderately and severely depressed participants.

### Symptom network analysis

The symptom scores for all the participants in the test set can also be represented as a correlation graph, a representation that is in line with the SNA approach. In our case, we can test whether the graph with predicted values is structurally equivalent to the graph with the real scores. We followed the method by van Borkulo et al. [14]. We used a permutation-based hypothesis test where network structures are estimated with sparse, $L_1$ regularized partial correlations. The test is implemented in the NCT^4^ package for R.

Two hypotheses were tested: about the invariant network structure, and the invariant global strength [46]. For the invariant network structure, the null hypothesis is that given the connection strength matrices $A_1$ and $A_2$ for graphs $G_1$ and $G_2$, all edge weights in $A_1$ are identical to those in $A_2$. The test statistic *M* is the largest difference between all connection strengths. For invariant global strength, the null hypothesis states that the overall connectivity is the same across the two graphs. The test statistic is the distance *S* that is defined as:

13 $$\begin{aligned} S(G_1, G_2) = |\sum _{i,j \in V}|A_{1ij}| - \sum _{i,j \in V}|A_{2ij}|| \end{aligned}$$

where *V* is the set of nodes in networks $G_1$ and $G_2$. On the test set, the invariant network structure test results were in *M* = 0.3648 and *p* value = 0.75, and the invariant global strength test in *S* = 0.0307 and *p* value = 0.96. Thus, we accept the null hypotheses of both tests and conclude that the symptom networks with predicted and real symptom scores are, indeed, structurally equivalent.

## Discussion

In this work, we address the automatic prediction of depression based on text transcripts. Instead of predicting the binary diagnostic label, as has been common in previous works, we propose to predict the fine-grained profile of symptoms that underlie the diagnosis of depression. According to our knowledge, such symptom-based approach has not been attempted before on the DAIC-WOZ data set, which has been used in many previous studies to develop clinical prediction models for depression. The predicted PHQ-8 symptom scores can be easily represented in various ways: as a total sum score representing the overall depression severity, and as both binary diagnostic and multi-class severity categories, thus also allowing for comparison with other systems. The experimental results showed that the symptom prediction approach is relatively robust and is on par with the previously published systems while at the same time giving a fine-grained overview of the person’s symptoms that the previous automatic diagnostic classification systems lack.

The models were able to predict some symptoms better than the others. In particular, Table 4 shows that such symptoms as lack of interest, depressed mood, feeling of being a failure, and feeling tired are among the most accurately predicted symptoms. This reflects the nature of the DAIC-WOZ data since these topics are discussed the most during each interview. Some of the symptoms may be addressed directly, e.g., by asking if the person was diagnosed with depression or PTSD in the past. The other symptoms are given attention as well, even though they are less direct, e.g., assessing the feeling of being a failure by asking what the interviewee’s friends and family think about them. The sleep-related symptom is also predicted relatively accurately; there are indeed questions about the person’s sleep problems, but they are not present in every interview. Finally, the symptoms related to eating, problems with concentration, and slowed down or overly agitated movement are not detected accurately by the model. Interestingly, the results show a RRMSE score close to 1 for these symptoms, which can indicate that there is little textual evidence of these symptoms in the data and thus, the model just learns an average score for these symptoms across the training data set.

The radar plots on Fig. 3 showed that the model’s predictions are close to the real ones for people with the depressive symptoms in the mild, and moderate severity range. However, the model tends to overevaluate the cases in the absent severity range and underevaluate the cases in the moderately severe and severe range. The underevaluation in the high range can be explained by the lack of data in this region: only seven interviews are available for training for the moderately severe subclass and four for severe one and even less for testing, with seven and two interviews, respectively. Additionally, Fig. 3 shows that the moving-related symptom consistently receives low scores across the whole depression severity spectrum. This is also reflected in the interviews; the moving-related symptoms are almost never verbally discussed, which can explain the high RRMSE score. We believe that the indicators of this symptom are mostly non-verbal; thus, a multi-modal setting that includes visual input might improve the results.

Interpreting the model predictions may help to understand the data itself better. To gain an understanding of the model’s decision-making, we extracted the saliency maps that track the prediction of each symptom back to the inputs, which in our case, are the dialogue turns. Saliency mapping is a gradient-based method that computes the importance of an input feature, i.e., an interview turn, based on the first-order derivative with respect to that feature [47]. Although saliency maps can be noisy [48], they can still provide useful information. They are also relatively easy to extract from most neural architectures. We extracted saliency maps for each symptom prediction and observed that the areas corresponding to the high importance are almost identical for each symptom and point to the area close to the middle of the interview. Indeed, each interview is structured in a way that the interviewer asks general non-depression-related questions in the beginning in order to establish trust with the interviewee. Similarly, at the end of the interview, the interviewer moves away from depression-related topics to wind the interviewee down.

**Fig. 4:**
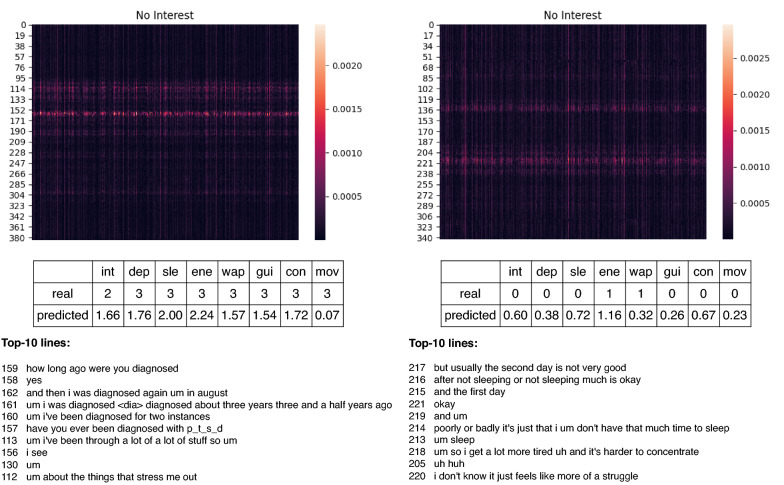
Saliency maps showing which parts of the interview are used for symptom predictions

Figure 4 shows an enhanced view of the gradients tracked back from the same symptom (lack of interest) to the input features for two different persons in the test set. The lines to which the highest absolute gradient value was attributed are “diagnostic”-related in the case of the person with a high PHQ-8 score indicating severe depressive symptoms (left in Fig.  4); for the non-depressed person (in the right), the model attributed high importance to the sleep-related utterances. After having studied the feature attributions across the whole test set, we observe that the model assigns importance to the symptom-related turns of the interview most of the time.

Every interview also includes the question “Have you been diagnosed with depression?”. Thus, it is plausible that the model can extract information relevant to predictions only from the answer to this question, thus using it as a shortcut. Although inspecting the saliency scores showed that the turn involving this question was not among the most important ones for most of the interviews, we investigated more thoroughly whether this question strongly correlates with the model’s predictions. First, we classified the answers to this question into three categories: “yes”, “no”, and “other.” “Yes” and “no” categories were assigned to the answers that can be clearly interpreted as positive or negative. If a participant tried to avoid the question or started to give extra information about their condition, the answer was classified as “other”. Fisher’s exact test at the *p* value $< 0.05$ was used to decide whether the depressed and non-depressed participant groups were different in their “yes” and “no” answers to this question. Similar analyses were conducted for every symptom with the groups formed by the symptom scores. Based on these analyses, we can conclude that the answers to the question “Have you been diagnosed with depression?” differ significantly between the groups formed based on different symptom scores, and thus, the model is suspect in utilizing these differences when making predictions. To estimate how dependent the model is on these answers, we replaced all the “yes” answers with a random answer variation from the “no” answer set and vice versa. Additionally, we replaced each “other” answer with another random answer from the “other” answer set as well. The same model was run on this perturbed test set, showing no drop in the $miF_1$ score (− 0.00%) and an insignificant minor drop in the $maF_1$ score (− 0.52%). Similar pattern was observed for $\text {MAE}$ (+ 0.06) and $ma\text {MAE}$ (+ 0.11). Thus, we can conclude that the model did not use this question with its explicit answers as a shortcut for making complex predictions.

This work also has several limitations. First, our work is limited to the DAIC-WOZ data set, which is, to our knowledge, the only high-quality data set that is easily obtainable from its authors. This data set is, however, quite small which might lead the models to overfit; nonetheless, the comparison of the development and test set results showed that the symptom-based model is fairly robust to overfitting. The data set also has a quite rigid structure, as all interview prompts are sampled from a closed set of prompts. Thus, we cannot assume the generalizability of the presented results to other data sets, which limits the applicability of our model. Furthermore, the transcribed interviews are long and require using a hierarchical architecture as one way to encode them. This entails a lot of computational power for training such a model due to its high computational complexity, thus limiting us in the choice of pre-trained contextual embeddings that are a foundation of most of the NLP neural architectures.

The main motivation for predicting symptoms instead of binary diagnostic classes, total depression severity or discrete severity class as has been custom in previous works, is the understanding of the need to keep up with advances in psychiatry, which moves towards more dimensional and descriptive diagnostic profiles. In our work, we also followed the general ideas of symptom network analysis and conducted analyses on the graphs of predicted and real symptom relations. However, because our data is cross-sectional, we are constrained to correlational analyses, whereas the real aim and strength of symptom network analysis rely on following the causal relations between symptoms, e.g., which symptoms cause which other symptoms. However, modeling these complex causal relationships with predictive models would require longitudinal data with several points of measurement in time.

## Conclusions

The main contribution of this work is highlighting the importance of keeping up with the advances in psychiatry and clinical psychology in the computational modeling and automatic prediction domain by moving away from predicting static diagnostic categories that contain limited information towards more descriptive and personalized symptom profiles. Towards this goal, we trained a multi-target hierarchical regression model to predict the severity scores of individual depression symptoms from patient–psychiatrist interview transcripts from the DAIC-WOZ corpus. The model achieved a mean absolute error (MAE) from 0.438 to 0.830 on eight depression symptoms and showed state-of-the-art results in binary depression estimation (0.739 $maF_1$) and total depression score prediction (3.78 MAE). Moreover, our model produced a symptom correlation graph that is structurally identical to the real one based on the static data. The applicability of the presented model is limited because it was trained and evaluated on the relatively small DAIC-WOZ data set. Despite this limitation, we believe that the proposed symptom-based approach should be developed further as it provides more in-depth information about the depressive condition than a general binary diagnosis. Moreover, it aligns with the symptom network analysis which is a recently proposed diagnostic approach in psychiatry.

## Data Availability

The DAIC-WOZ data set that supports the findings of this study is available from The University of Southern California Institute for Creative Technologies (https://dcapswoz.ict.usc.edu/) but restrictions apply to the availability of these data, which were used under license for the current study, and so are not publicly available. Data set is, however, available from the University of Southern California Institute for Creative Technologies upon reasonable request [22]. The code of the model is available in the author’s repository: https://git.unicaen.fr/kirill.milintsevich/hierarchical-depression-symptom-classifier.

## Abbreviations

BERT: Bidirectional encoder representations from transformers
DAIC-WOZ: Distress analysis interview corpus wizard-of-oz
DSM-5: Diagnostic and statistical manual of mental disorders
GNN: Graph neural network
GPC: General psychotherapy corpus
HCAN: Hierarchical contextual attention network
HRSD: Hamilton rating scale for depression
MAE: Mean absolute error
MDD: Major depressive disorder
MLM: Masked language modeling
NCT: Network comparison test
NLP: Natural language processing
PHQ: Patient health questionnaire
RRMSE: Relative root mean square error
SNA: Symptom network analysis

## Footnotes

1 This requires a longitudinal analysis over time.

2 Our contacts with Alexander Street Press were unfruitful to get the GPC corpus.

3 https://huggingface.co/sentence-transformers/all-distilroberta-v1.

4 https://cran.r-project.org/web/packages/NetworkComparisonTest/.
